# Bilateral Intracranial Vertebral Artery Stenosis Presenting as Recurrent Prolonged Presyncopal Episodes

**DOI:** 10.1155/2015/251536

**Published:** 2015-09-01

**Authors:** Banu Sundar, Majaz Moonis, Ajay Wakhloo, Ajit Puri

**Affiliations:** University of Massachusetts Medical School, Worcester, MA 01655, USA

## Abstract

Amongst various mechanisms of presyncopal events, posterior circulation disease needs to be considered. This particular mechanism has been underrecognized. We describe a case of a 76-year-old patient with recurrent posterior circulation TIAs, presenting as recurrent prolonged presyncopal events.

## 1. Background

20% of all ischemic strokes occur in the posterior circulation, and these strokes may have varied presentation [1]. Posterior circulation ischemia rarely causes only one symptom but rather produces a collection of symptoms and signs, depending on which area is ischemic [2]. Symptoms may range from decreased level of consciousness to coma, often associated with signs of long tract and multiple cranial nerve involvement.

The commonest clinical pattern in patients with bilateral Intracranial Vertebral Artery (ICVA) disease is presence of multiple Transient Ischemic Attacks (TIAs). These episodes are often position-sensitive and may continue for months or even years [3].

We present a unique case of posterior circulation TIAs, manifesting as isolated, recurrent, unprovoked, nonpositional, presyncopal episodes, with bilateral ICVA stenosis. To the best of our knowledge, no similar case has been reported elsewhere in the literature.

## 2. Case Presentation

A 76-year-old patient, with past medical history of hypertension and hyperlipidemia, presented to the emergency room (ER) with a presyncopal episode, described as a feeling of passing out, almost collapsing, and being light-headed, without loss of consciousness. This episode started one week prior to the ER visit. There was no associated nausea, vomiting, diplopia, weakness, or dysphagia. During the entire hospitalization, the patient felt light-headed. On examination, the patient did not have orthostatic hypotension and had full conjugate eye movements without nystagmus or other cranial nerve involvement. There were no motor or sensory deficits. However, the patient felt unsteady while walking.

**Figure 1:**
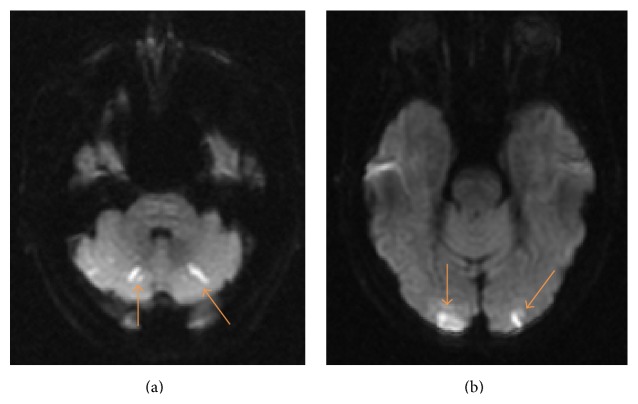
Acute ischemic infarct, MRI brain DWI sequences. (a) Bilateral cerebellum. (b) Bilateral occipital lobes.

Brain magnetic resonance imaging (MRI) on this admission showed multiple acute infarctions, in the bilateral cerebellar hemispheres (Figure 1(a)) and the parietal and occipital lobes. Brain magnetic resonance angiography (MRA) revealed focal high-grade segmental stenosis of the intradural segment of the left vertebral artery (LVA), just proximal to the origin of the Posterior Inferior Cerebellar Artery (PICA). In addition, the MRA brain showed a short segment high-grade stenosis of the intracranial segment of the right vertebral artery (RVA). MRA of the neck showed no hemodynamically significant stenosis of bilateral common carotid, internal carotid, and vertebral arteries in the neck.

The dizziness resolved with hydration. The patient was discharged on aspirin and high dose statins.

Six months later, the patient had recurrent episodes of similar lightheadedness, but this time accompanied by bilateral facial paresthesias that required rehospitalization. Neurological examination showed no focal deficits. These symptoms were fleeting and resolved shortly after hospitalization. Repeat MRI brain showed multiple foci of new restricted diffusion in the bilateral occipital (Figure 1(b)) and right temporal lobes. During this hospitalization, aspirin was changed to Clopidogrel. On discharge, the patient was continued on best medical management for secondary prevention of cerebrovascular disease.

Other investigations completed during this hospitalization include the following: ESR 13; CRP 2.1; ECHO; EF 55–60%, no Right-Left shunt; normal left atrial size; normal left ventricular ejection fraction and diastolic function; and 30-day event monitor, during which patient experienced presyncopal episodes and showed no arrhythmias.

Subsequently, after four months, the patient had several ER visits over a two-month period. During these visits, the patient presented with presyncopal events, which were described as “a feeling of near collapse.” There was no loss of consciousness associated with any of these episodes. This was again similar to the prior presyncopal episodes, but without any neighborhood symptoms or signs. Comprehensive neurological examinations during these ER visits were normal. MRI of the brain demonstrated no new acute stroke. CT angiogram of head and neck showed unchanged ICVA stenosis, without hemodynamically significant stenosis of ECVAs and carotid arteries.

Following these ER visits, the patient continued to experience disabling presyncopal episodes on a daily basis. Episodes were unprovoked and not associated with orthostatic hypotension. The patient was adequately hydrated during these episodes. Possibility of posterior circulation TIA was considered. Referral to the neurointerventional service was made to confirm the degree of stenosis of the vertebral arteries (VA).

**Figure 2:**
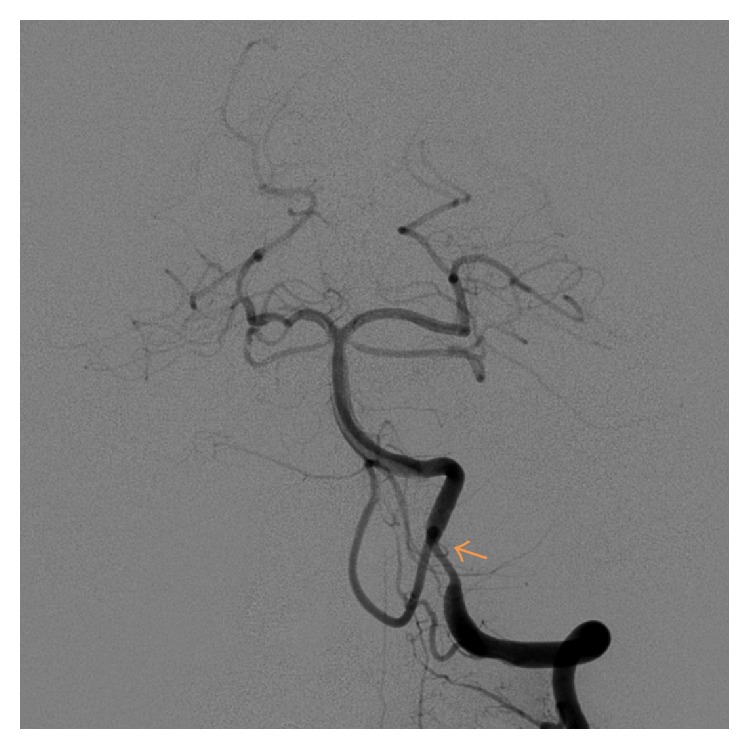
Left vertebral artery angiogram demonstrating stenosis (arrow).

The patient underwent a 4-vessel cerebral angiogram, which showed mild to moderate narrowing at the junction of the right V3-V4 segments, proximal to the origin of the right PICA, and moderate to severe angiographic (approximately 65%) narrowing at the junction of the left V3-V4 segments (Figure 2), proximal to the origin of the left PICA. There was no hemodynamically significant stenosis of the carotid arteries.

The patient underwent angioplasty and stenting of the LVA. Following the procedure, the patient's symptoms resolved. To this date, the patient has been free of the presyncopal episodes. A follow-up one-year angiogram showed no restenosis of the LVA and unchanged stenosis of the right ICVA.

## 3. Discussion

Symptoms and signs of posterior circulation strokes vary in frequency and presentation, according to localization related to the proximal, middle, or distal intracranial posterior circulation territory. Proximal intracranial posterior circulation supply consists of branches of distal ICVA, PICA. Middle intracranial posterior circulation is supplied by Anterior Inferior Cerebellar Artery (AICA), penetrating branches of Basilar Artery (BA), and superior cerebellar artery (SCA). Distal intracranial posterior circulation is supplied by SCA and Posterior Cerebral Artery (PCA) [4–6].

The most frequent symptom during TIAs owing to ECVA disease and V1 lesions is reported to be dizziness [5, 7, 8]. However, even in these cases, there were associations such as veering to one side, gait ataxia, visual blurring, and perioral paresthesias [6]. Our patient's presentation of posterior circulation TIA is unique, as it consists of recurrent, isolated, prolonged dizziness, in association with bilateral ICVA disease with no ECVA disease.

Vertebrobasilar (VB) stenosis is associated with high risk of recurrent TIA or stroke. This risk is particularly higher within the first months from the initial event [1, 9]. In line with literature, our patient sustained recurrent stroke in a six-month interval, on both occasions. Both strokes were in distal intracranial posterior circulation, in SCA and PCA territory. This was in the setting of unchanged stenosis of both ICVAs over the course of several months.

**Figure 3:**
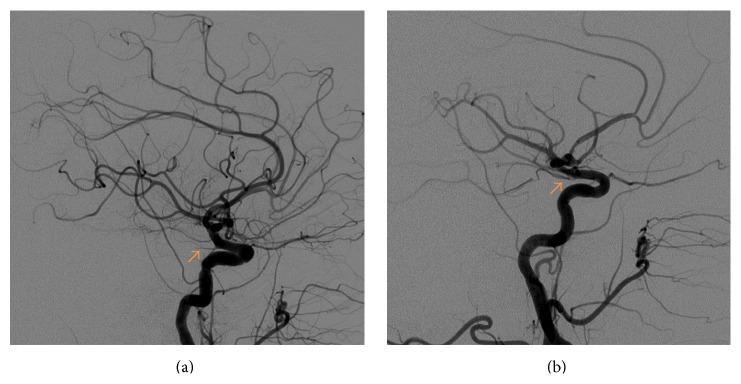
RICA (a) and LICA (b) demonstrating small-sized posterior communicating arteries (arrows).

Most patients with bilateral ICVA disease were reported to have good collaterals from the anterior circulation through the posterior communicating arteries, the posterior circulation through the superior cerebellar arteries, or both [10]. After the second stroke, our patient sustained recurrent, presyncopal episodes. These hemodynamic spells were without new ischemic changes or progression of the bilateral ICVA disease. The most likely explanation is the poor collaterals from the bilateral posterior communicating arteries in the setting of the bilateral ICVA stenosis (Figures 3(a) and 3(b)).

**Figure 4:**
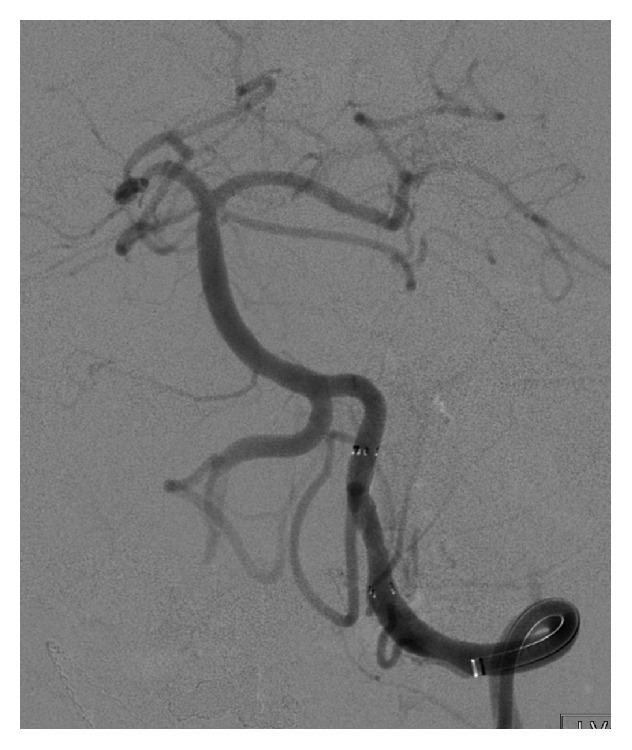
Left vertebral artery angiogram image demonstrating successful stenting.

In patients with intracranial arterial stenosis, based on the Stenting and Aggressive Medical Management for Preventing Recurrent Stroke in Intracranial Stenosis (SAMMPRIS) trial results, aggressive medical management was superior to Percutaneous Transluminal Angioplasty and Stenting (PTAS), because the risk of early stroke after PTAS was reported to be high, and risk of stroke with aggressive medical therapy alone was lower than expected [11]. Our patient continued with disabling symptoms, in spite of maximizing the medical management. These symptoms resolved after LVA artery stenting (Figure 4) and angioplasty.

In conclusion, our patient's clinical scenario appears unique with respect to the recurrent hemodynamic spells, without other symptoms/signs of posterior circulation ischemia and failure of the best medical management to resolve these symptoms. The resolution of presyncopal episodes after intervention suggests the importance of vascular imaging in similar patients without other obvious causes of syncope and carefully selecting patients who might benefit from interventional procedures after failed best medical management.
